# Responses to dietary levels of methionine in broilers medicated or vaccinated against coccidia under *Eimeria tenella*-challenged condition

**DOI:** 10.1186/s12917-018-1470-8

**Published:** 2018-04-25

**Authors:** Anqiang Lai, Guozhong Dong, Daijun Song, Tan Yang, Xiaolong Zhang

**Affiliations:** 1 College of Animal Science and Technology, Southwest University, Beibei, Chongqing, 400716 People’s Republic of China; 2 Sichuan Giant Star Company’s Experimental Station, Leshan, Sichuan 614800 People’s Republic of China

**Keywords:** Methionine, Anticoccidial drug, Vaccination, *E. tenella*, Broilers

## Abstract

**Background:**

Coccidiosis is a prevalent problem in chicken production. Dietary addition of coccidiostats and vaccination are two approaches used to suppress coccidia in the practical production. Methionine (Met) is usually the first limiting amino acid that plays important roles in protein metabolism and immune functions in chickens. The present study is aimed to investigate whether increasing dietary Met levels will improve the anticoccidial effects in broilers medicated or vaccinated against coccidia under *Eimeria (E.) tenella*-challenged condition. Two thousand male Partridge Shank broiler chicks were obtained from a hatchery. After hatch, birds were weighed, color-marked and allocated equally into two anticoccidial treatments, namely medicated and vaccinated groups. Chicks were either fed, from 1 d of age, diets containing coccidiostat (narasin) or diets without the coccidiostat but were inoculated with an anticoccidial vaccine at 3 d of age. At 22 d of age, 1080 chicks among them were randomly allocated evenly into 6 groups under a 2 × 3 treatment with 2 anticoccidial programs and 3 dietary methionine (Met) levels. Chicks medicated or vaccinated against coccidia were fed diets containing 0.45%, 0.56% or 0.68% of Met from 22 to 42 d of age. All chicks were orally introduced with an amount of 5 × 10^4^ sporulated oocysts of *E. tenella* at 24 d of age. The growth performance, serum anti-oxidative indexes, intestinal morphology, cecal lesion scores, fecal oocyst counts and immune parameters were measured.

**Results:**

The results showed increasing dietary Met level from 0.45% to 0.56% and 0.68% improved weight gain and feed conversion of broilers medicated against coccidia. In contrast, higher dietary levels of Met did not improve growth performance of the vaccinated chickens. Higher Met levels helped the medicated chickens resist *E. tenella* infection, as indicated by improved intestinal morphology and immune functions as well as decreased cecal lesion and fecal oocyst counts.

**Conclusions:**

Anticoccidial vaccination is a better strategy for controlling coccidiosis than feeding narasin, due to not only greater growth performance, but also the lower Met supplementation. Furthermore, higher dietary Met levels improved growth performance of chickens medicated rather than vaccinated against coccidia under *E. tenella*-challenged condition.

## Background

Methionine (Met) is the first limiting amino acid in broiler chickens fed maize-soybean diets [1]. Supplementation with Met in the diets is usually necessary to ensure the proper functions in protein metabolism and immune function. Met, like other amino acids, is the component of tissue proteins, thus serves as substrates for protein synthesis. In addition, Met participates in the methyl group metabolism, which includes both transmethylation and remethylation pathways and the transsulfuration pathway [2, 3]. Biological methylation of DNA, RNA, and protein is a critical approach for both pre-transcriptional and post-transcriptional regulations [4–6]. Through the transsulfuration, Met serves as a precursor for the synthesis of other sulfur amino acids, notably cystine, and several other metabolites, including glutathione (GSH), coenzyme A, taurine and inorganic sulfur [3]. GSH and taurine are essential compounds for host defense against oxidative stress. Met is also known to play critical roles in both cellular and humoral immunities of chickens. Dietary Met is involved in antibody production and cell-mediated immune responses in broilers [7]. Met deficiency can cause pathological and ultrastructural changes of thymus, reduce serum interlukin-2 (IL-2) contents and the T-cell population, and induce impaired proliferative function of T-cells and increased percentage of apoptotic cells in spleen [1]. There is evidence that Met metabolite, GSH, regulates nuclear transcription factor κB pathway, T-helper cell function and antibody and interferon-γ (IFN-γ) production in response to immunological challenges [7]. Therefore, the involvement of Met in immune function is probably related to the synthesis of antibodies, cytokines and other cytotoxic substances as well as the regulation of gene expression, signaling pathways and cellular redox state [7].

Coccidiosis, caused by several species of *Eimeria*, is prevalent in the poultry industry and continues to cause the most severe health and welfare problems. Malabsorption and diarrhea are typical symptoms of coccidiosis. Chickens with coccidiosis thus have depressed growth rate, impaired feed conversion rate, and, in worst cases, increased mortality [8]. Dietary addition of coccidiostats and vaccination against coccidia are two primary strategies for coccidiosis control, which effectively decrease the incidences and severity of coccidiosis [9]. Their protective effects against coccidial infection are supposed to ensure a normal gut function for the absorption of amino acids in diets.

However, both strategies have their shortcomings. The use of coccidiostats over the past 50 years has caused some degree of resistance of *Eimeria* strains, which conversely decreases anticoccidial effectiveness of drugs [9]. But to what extent drug-tolerant *Eimeria* strains influence the nutrient absorption of chicks remains unclear. Coccidial vaccination is generally associated with secondary enteritis, or even sporadically, necrotic enteritis incidence [9]. In addition, the immune regulatory actions of vaccines pose significant impact on the metabolism of certain amino acids, like Met. Amino acid metabolism is markedly altered to support the enhanced metabolic flows and cellular synthesis during the inflammatory and immune responses [10]. It has been found that the loss of urinary N is increased with the immune responses, which suggests an increased expenditure of endogenous amino acid sources [10]. The availability of amino acids influences aspects of the immune responses, which depend upon increased DNA and protein synthesis during lymphocyte replication and antibody synthesis. Previous research has suggested that Met supplemental level should be above the NRC (1994) recommendations under various catabolic conditions [11]. Maroufyan et al. [11] found that feeding Met at the level of twice NRC (1994) recommendation enhanced immune response in broiler chickens challenged by infectious bursal disease. Mirzaaghatabar et al. [2] discovered that feeding a diet containing 1.2% Met has improved humoral immunity and production performance of chickens compared to 0.45% Met. A study performed in various genotypes of broilers shows that antibody titers against antigens derived from sheep red blood cells were increased with the concentrations of Met from 3.91 to 5.54 g/kg in the diets [12].

However, the effects of dietary Met levels on the resistance to coccidia in broilers under different anticoccidial programs remain unclear. Therefore, this study is aimed to investigate whether increasing dietary Met levels will improve the anticoccidial effects and growth performance in broilers medicated or vaccinated against coccidia under *Eimeria (E.) tenella*-challenged condition.

## Methods

### Experimental design and procedures

Two thousand male Partridge Shank broiler chicks were obtained from a hatchery in which eggs were set and hatched in a common incubator. After hatch, birds were weighed, color-marked and allocated equally into two anticoccidial treatments, namely medicated and vaccinated groups. In the medicated group, chicks were fed diets always containing 60 mg/kg narasin (an anticoccidial drug) in the starter diet and the grower diet, whereas chicks in the vaccinated group were inoculated with an anticoccidial live vaccine (1 dose per bird, 7-valent anticoccidial live vaccine, Schering-Plough, Shanghai, China) at 3 d of age without dietary addition of anticoccidial drugs. All chicks received a complete starter diet (Table 1) that meets the Feeding Standard of Chickens [13]. Feed and water were available to the birds ad libitum. For the first 7 days, the chicks were housed in stainless steel battery cages (68 cm × 70 cm, five birds/cage) in a temperature-controlled house with continuous lighting. The temperature of the room was maintained at 32 to 34 °C for the first 3 d and then reduced by 2 to 3 °C per week to a final temperature of 20 °C. Birds were protected against Newcastle disease, infectious bursal disease, avian influenza and fowl pox according to the routine vaccination schedule. Chicks were transferred at 7 d of age into floor pens with free water and feed access as well as constant illumination. The floor of each pen was bedded with rice hull. From 22 to 42 d of age, 1080 chicks among them were randomly allocated evenly into 6 groups under a 2 × 3 treatment with 2 anticoccidial programs and 3 dietary Met levels. Chicks medicated or vaccinated against coccidia were fed diets containing 0.45%, 0.56 or 0.68% of Met from 22 to 42 d (Table 2). These dietary Met levels are equivalent to 100%, 125% and 150% of the growing broiler requirement, respectively [13]. Each experimental treatment was replicated in six separate pens with 30 chicks per pen. All chicks were orally introduced with an amount of 5 × 10^4^ sporulated oocysts of *E. tenella* at 24 d of age.

**Table 1:** Composition and nutrient levels (air-dry basis, %) of the starter diet (1–21 d of age)

Ingredient	Content
Corn	55.04
Wheat	5.00
Dehulled soybean meal	35.00
Soybean oil	1.30
Dicalcium phosphate	1.85
Limestone	1.16
Choline chloride	0.10
Salt	0.32
L-lysine·HCl	0.03
Vitamin Premix^a^	0.10
Trace-element premix^b^	0.10
Total	100
Nutrient level^c^	Content
ME (Mcal/kg)	2.90
Crude protein	21.00
Lysine	1.07
Methionine	0.31
Methionine+cystine	0.68
Threonine	0.76
Calcium	1.00
Available phosphorus	0.45

^a^Provided per kg of diet: vitamain A, 8000 IU; vitamain D_3_,2000 IU; vitamain E,15 IU; vitamain K_3_, 1.5 mg; thiamine, 2 mg; riboflavin, 5 mg; pyridoxine, 5 mg; vitamain B_12_, 0.02 mg; folic acid, 0.7 mg; nicotinic acid, 40 mg; pantothenic acid, 12 mg; biotin, 0.2 mg

^b^Provided per kg of diet: copper, 10 mg (as copper sulfate); iron, 90 mg (as ferrous sulfate); manganese, 100 mg (as manganese sulfate); zinc, 100 mg (as zinc sulfate); selenium, 0.3 mg (as sodium selenite); iodine, 0.5 mg (as calcium iodate)

^c^ME is metabolizable energy and is a calculated value. Other nutrient levels are measured values

**Table 2:** Composition and nutrient levels (air-dry basis, %) of grower diets (22–42 d of age)

	Medicated groups^d^			Vaccinated groups		
Met concentrations:	0.45%	0.56%	0.68%	0.45%	0.56%	0.68%
Ingredients						
Corn	60.41	60.29	60.05	60.41	60.29	60.05
Wheat	5.00	5.00	5.00	5.00	5.00	5.00
Dehulled soybean meal	28.50	28.50	28.50	28.50	28.50	28.50
Soybean oil	2.50	2.60	2.70	2.50	2.60	2.70
Dicalcium phosphate	1.60	1.60	1.60	1.60	1.60	1.60
Limestone	1.20	1.20	1.20	1.20	1.20	1.20
L-Lysine·HCl	0.03	0.03	0.03	0.03	0.03	0.03
Choline chloride	0.10	0.10	0.10	0.10	0.10	0.10
Salt	0.31	0.31	0.31	0.31	0.31	0.31
Vitamin Premix^a^	0.10	0.10	0.10	0.10	0.10	0.10-
Trace-element premix^b^	0.10	0.10	0.10	0.10	0.10	0.10
DL-methionine	0.15	0.27	0.39	0.15	0.27	0.39
Total	100	100	100	100	100	100
Nutrient levels^c^						
ME (Mcal/kg)	3.00	3.00	3.00	3.00	3.00	3.00
Crude protein	18.50	18.50	18.50	18.50	18.50	18.50
Methionine	0.45	0.56	0.68	0.45	0.56	0.68
Lysine	1.00	1.00	1.00	1.00	1.00	1.00
Threonine	0.65	0.65	0.65	0.65	0.65	0.65
Calcium	0.95	0.95	0.95	0.95	0.95	0.95
Available phosphorus	0.40	0.40	0.40	0.40	0.40	0.40

^a^Provided per kg of diet: vitamain A, 8000 IU; vitamain D_3_,2000 IU; vitamain E,15 IU; vitamain K_3_, 1.5 mg; thiamine, 2 mg; riboflavin, 5 mg; pyridoxine, 5 mg; vitamain B_12_, 0.02 mg; folic acid, 0.7 mg; nicotinic acid, 40 mg; pantothenic acid, 12 mg; biotin, 0.2 mg

^b^Provided per kg of diet: copper, 10 mg (as copper sulfate); iron, 90 mg (as ferrous sulfate); manganese, 100 mg (as manganese sulfate); zinc, 100 mg (as zinc sulfate); selenium, 0.3 mg (as sodium selenite); iodine, 0.5 mg (as calcium iodate)

^c^ME is metabolizable energy and is a calculated value. Other nutrient levels are measured values

^d^Ananticoccidial drug (narasin, 60 mg/kg diet) was added to diets in medicated groups, and an anticoccidial 7-valent live vaccine was inoculated in vaccinated groups

### Performance measurements and fecal oocyst counts

Average daily gain (ADG), average daily feed intake (ADFI) and feed to gain ratio (F/G) were measured through d 22 to d 42. Mortality data were collected and recorded on a daily basis, and the mortality was low in all treatment groups. Excreta was observed daily after oocyst challenge and oocyst counts were determined in excreta samples taken from each pen on d 6 and d 7 after challenge. Oocysts population was enumerated by microscopy according to the procedure of Long and Joyner [14]. The final oocyst counts were the average of those on d 6 and d 7 after challenge.

### Blood and intestinal sample collection and processing

At 42 d of age, 30 birds per treatment were randomly selected (6 pens per treatment and 5 birds from each pen) after feed deprivation for 12 h. Blood samples (about 5 mL each) were taken from the wing vein and centrifuged at 3000×g for 15 min at 4 °C to separate serum, which was frozen at − 20 °C for further analysis. Blood samples for testing CD4^+^ and CD8^+^ lymphocyte populations (about 5 ml each), which were taken from wing vein, were stored in EDTA anticoagulation tubes. After blood collection, birds were killed by cervical dislocation. The ileum and cecum were separated and excised for histological examination. Intestinal contents from the cecum were dissolved into 0.9% NaCl solution (1:1, *v*/v) and then centrifuged at 10000×g for 1 min. The supernatant was used to detect the concentration of secreted immunoglobulin A (sIgA) in the lumen. The cecal tonsils were separated and stored in liquid nitrogen until Reverse transcription-PCR (RT-PCR) analysis.

### Histological examination

The lesion scores of the cecum were determined and recorded according to the method of Johnson et al. [15]. The lesion scores range from 0 (no gross lesion) to 4 (most severe gross lesion). One-cm section of ileum was fixed in 4% paraformaldehyde solution, dehydrated and embedded in paraffin. The section was cut 4 μm long and stained with hematoxylin-eosin. The villus length (VH), crypt depth (CD), and VH to CD ratio (V/C) were measured by a Nikon phase contrast microscope coupled with a Micro Comp integrated digital imaging analysis system (Nikon Eclipse 80i, Nikon Corp., Tokyo, Japan).

### Enzyme-linked immunosorbent assay (ELISA)

sIgA levels in cecal lumina were examined using the commercially available ELISA kit (Sigma, St. Louis, MO, USA) following the manufacture protocols. Briefly, a 96 well coated with one specific antibody at the bottom was incubated at the room temperature. After washing, suitably diluted samples were added to designated wells with subsequent addition of secondary antibodies and ortho-phenylenediamine. Following an incubation period, the reaction was stopped with H_2_SO_4_ and the absorbance was determined at 492 nm in Multiskan ELISA plate reader (Thermolabsystems, Finland).

### RT-PCR assays

Total RNA was isolated from the cecal tonsil tissues using TRIzol Reagent (TaKaRa, Dalian, Liaoning, China). Reverse transcription of total RNA was completed using Prime Script RT Reagent Kit (TaKaRa). Primers for RT-PCR were presented in Table 3. PCR was performed with a 7500 Fast Real-Time PCR System (Applied Biosystems, MA, USA), and data were analyzed with the 2^-∆∆CT^ method. β-tubulin expression was used as an internal control to calculate the relative expression levels of targeted genes (Table 3). The mRNA level of each targeted gene for the control birds was assigned a value of one.

**Table 3:** Sequences of primers for the PCR assay

Gene^a^	Forward(5′-3′)	Reverse(5′-3′)
*IL-2*	TCTTTGGCTGTATTTCGGTAG	CTTTATTAATGTTTGAAGAGGTTTC
*IFN-γ*	AGCCGCACATCAAACACAT	TCACCTTCTTCACGCCATC
*TNF-α*	CTTGGCTGTCGTGTGGTGG	CGTGGGCATTGCAATTTGG
*β-tubulin*	GCGTTATTTGACTGTGGCG	GGAGCTGTTCTTGGTCTGGA

^a^*IL-2*: interleukin-2; *IFN-γ:* interferon-γ; *TNF-α*: tumor necrosis factor-α

### Flow cytometry

Flow cytometry was used to assay CD4^+^ and CD8^+^ lymphocyte populations in peripheral blood. The fluorescein isothiocyanate (FITC)-CD4^+^ and phycoerythrin (PE)-CD8^+^ mouse anti-chicken antibodies (SouthernBiotech, Birmingham, USA) and blood samples were added to each tube and mixed by gentle shaking at 4 °C for 30 min. Erythrocytes were then lysed with 2 mL of Lysing Solution (Becton Dickinson (BD), Franklin Lakes, NJ, USA) at room temperature for 20 min. The lysate was centrifuged at 1500×g for 5 min and the supernatant was removed. The pellet was resuspended in phosphate-buffered saline (PBS). The lysate was centrifuged again at 1500×g for 5 min, the supernatant was removed, and the pellet was resuspended in 500 mL of PBS for assay performed on a flow cytometer (Coulter XL FCM, Beckman Coulter, Brea, CA, USA).

### Measurements of anti-oxidative indexes

GHS peroxidase (GPx) in the serum was tested using detection kits (Beyotime Biotechnology, Shanghai, China), according to the manufacturer’s instructions. Total antioxidant capacity (T-AOC) of the serum was measured using the azino-diethyl-benzthiazoline sulfate (ABTS) method. Incubation of ABTS with H_2_O_2_ and a peroxidase (metmyoglobin) results in the production of the blue-green radical cation ABTS^+^. Antioxidants in the serum suppress this color production proportionally to their concentration. The system was standardized using Trolox, a water-soluble vitamin E analogue. The results were expressed as mmol Trolox equivalent. In malondialdehyde (MDA) assay, 2 mL 0.6% thiobarbituric acid was added to 2 mL of the serum in a 10 mL tube. This tube was next kept in boiling water for 15 min and placed on ice to cool down, before the optical density was measured at 532 nm. The results were expressed as nmol MDA/mL.

### Statistical analysis

Statistical analyses were performed using the general linear procedure of the SAS software (Version 9.1; SAS Institute Inc., Cary, NC, USA). A two (anticoccidial programs) by three (dietary Met levels) factorial arrangement of treatments in a randomized complete block design was employed in the analysis of variance. All data were analyzed using anticoccidial programs, dietary Met levels, and their interactions as main effects. Differences among treatment means were classified by Duncan’s multiple range test and significant difference was defined at *P* < 0.05. Results are reported as mean ± standard deviation.

## Results

### Growth performance of chickens

Under *E. tenella*-challenged conditions, anticoccidial programs and dietary Met levels had significant effects on final weight, ADG and ADFI (*P* < 0.05 or 0.01; Table 4). F/G ratio was influenced only by dietary Met levels (*P* < 0.05). There were interactions between anticoccidial programs and dietary Met levels for final weight, ADG and F/G ratio (*P* < 0.01). With regard to the anticoccidial programs, the vaccinated chicks displayed better or identical performance than the medicated chicks except for the high diet Met level (0.68%). In regard to dietary Met levels, feeding 0.45% Met to medicated chicks and 0.68% Met to vaccinated chicks resulted in the lowest final weight, and medicated chickens showed increased ADG and decreased F/G ratio with increasing dietary Met concentrations from 0.45 through to 0.68% (*P* < 0.05). For medicated chickens, high supplemental levels of Met had no effects on ADFI (*P* > 0.05). In contrast, feeding 0.68% Met to vaccinated chickens decreased final weight, ADG and ADFI compared to 0.45% and 0.56% Met (*P* < 0.05). Increasing dietary levels of Met had no beneficial effects on F/G ratio in vaccinated chickens (*P* > 0.05).

**Table 4:** Growth performance of chickens after indicated treatments^1^

	Medicated groups			Vaccinated groups			*P* value		
Met^2^ concentrations:	0.45%	0.56%	0.68%	0.45%	0.56%	0.68%	Anticoccidial program	Met levels	Anticoccidial program × Met levels
Initial BW (g)	466 ± 12	470 ± 14	460 ± 8	470 ± 10	463 ± 11	466 ± 7	0.754	0.499	0.303
Final BW (g)	1187 ± 10^d^	1221 ± 19^bc^	1212 ± 12^c^	1244 ± 14^a^	1230 ± 0.17^b^	1196 ± 13^d^	0.004	0.009	<.001
ADFI (g/d)	87.23 ± 0.93^b^	87.53 ± 1.66^b^	86.69 ± 1.11^b^	89.42 ± 0.94^a^	89.47 ± 2.56^a^	86.47 ± 2.01^b^	0.027	0.014	0.166
ADG (g/d)	36.06 ± 0.32^c^	37.58 ± 0.94^b^	37.61 ± 0.76^b^	38.73 ± 0.93^a^	38.36 ± 1.11^ab^	36.50 ± 0.72^c^	0.008	0.035	<.001
F/G	2.42 ± 0.02^a^	2.33 ± 0.03^b^	2.31 ± 0.04^b^	2.31 ± 0.03^b^	2.33 ± 0.03^b^	2.37 ± 0.05^b^	0.188	0.032	<.001

^1^An anticoccidial drug (narasin, 60 mg/kg diet) was added to diets in medicated groups, and an anticoccidial 7-valent live vaccine was inoculated in vaccinated groups

^2^*Met* methionine, *BW* body weight, *ADFI* average daily feed intake, *ADG* average daily gain, *F/G* feed to gain ratio

^a,b,c,d^The data of the same row not sharing a lowercase letter differ (*P* < 0.05; *n* = 180)

### Intestinal morphology and the number of excreted coccidial oocysts

The VH, V/C, cecal lesion scores and excreted oocyst numbers were influenced by anticoccidial programs (*P* < 0.05 or 0.01; Table 5). Dietary Met levels changed the VH, CD, V/C, cecal lesion scores and excreted oocyst numbers (*P* < 0.05). For all these indexes, anticoccidial programs and dietary Met levels had an interaction (*P* < 0.05 or 0.01). Medicated chickens showed increased VH and V/C after feeding 0.56% and 0.68% Met compared to 0.45% Met (*P* < 0.05). The CD was decreased after medicated chickens were fed 0.56% and 0.68% Met (*P* < 0.05). However, vaccinated chickens had decreased VH after increasing Met levels (*P* < 0.05). Feeding high levels of Met had no effect on CD of vaccinated chickens (*P* > 0.05). Feeding 0.68% Met decreased V/C in vaccinated chickens (*P* < 0.05).

**Table 5:** Intestinal morphology and coccidian oocyst numbers after indicated treatments^1^

	Medicated groups			Vaccinated groups			*P* value		
Met^2^ concentrations:	0.45%	0.56%	0.68%	0.45%	0.56%	0.68%	Anticoccidial program	Met levels	Anticoccidial program × Met levels
VH (um)	845.73 ± 90.97^d^	907.96 ± 100.88^bc^	912.49 ± 111.38^b^	923.37 ± 102.59^a^	905.36 ± 100.60^bc^	899.85 ± 95.98^c^	0.027	0.033	0.009
CD (um)	124.17 ± 15.52^a^	112.64 ± 14.08^b^	109.84 ± 13.23^b^	113.91 ± 15.23^b^	112.21 ± 11.34^b^	113.15 ± 12.59^b^	0.069	0.040	0.039
V/C	6.90 ± 0.60^d^	8.09 ± 0.82b^c^	8.38 ± 1.07^a^	8.15 ± 0.78^b^	8.12 ± 0.98^b^	8.00 ± 0.90^c^	0.031	0.038	0.006
Score	0.89 ± 0.11^a^	0.75 ± 0.09^b^	0.68 ± 0.06^c^	0.46 ± 0.05^e^	0.48 ± 0.05^e^	0.59 ± 0.07^d^	0.008	0.019	0.006
ON (× 10^5^/g)	1.47 ± 0.18^a^	1.05 ± 0.13^b^	1.00 ± 0.09^b^	0.39 ± 0.02^c^	0.41 ± 0.04^c^	0.42 ± 0.05^c^	0.004	0.025	0.008

^1^An anticoccidial drug (narasin, 60 mg/kg diet) was added to diets in medicated groups, and an anticoccidial 7-valent live vaccine was inoculated in vaccinated groups

^2^*Met* methionine, *VH*, villus height, *CD*, crypt depth, *V/C* VH to CD ratio; Score: the lesion scores of the cecum; ON: oocyst output

^a,b,c,d,e^The data of the same row not sharing a lowercase letter differ (*P* < 0.05, *n* = 30)

Vaccinated chickens showed lower cecal lesion scores and excreted coccidial oocysts than medicated chickens, irrespective of Met levels in diet (Table 5). Medicated chickens fed 0.56 and 0.68% Met showed decreased cecal lesion scores (*P* < 0.05). But, vaccinated chickens fed 0.68% Met exhibited increased cecal lesion scores, compared with those fed 0.45% and 0.56% Met (*P* < 0.05). The number of excreted coccidial oocysts was decreased when medicated chickens were fed 0.56% and 0.68% Met (*P* < 0.05). High supplemental levels of Met in the diet did not affect the number of excreted coccidial oocysts in vaccinated chicks.

### Immune responses of chickens

Anticoccidial programs had no effects on the percentages of CD4^+^ cells in the blood and the mRNA expression of *IFN-γ* in the cecal tonsil tissue (*P* > 0.05), but influenced CD8^+^ cell percentages, CD4^+^/CD8^+^, cecal luminal sIgA levels and the mRNA expression of *TNF-α* and *IL-2* (*P* < 0.05 or 0.01; Table 6). Dietary Met levels impacted on all these indexes, and had interactions with anticoccidial programs for them (*P* < 0.05 or 0.01). The data showed that increasing dietary Met concentrations to 0.56% and 0.68% elevated the percentages of blood CD4^+^ cells in medicated chicks (*P* < 0.05). However, the increase in dietary Met concentrations decreased the CD4^+^ cell percentages in vaccinated chickens (*P* < 0.05). CD8^+^ cell percentages in medicated chickens were notably increased as well after feeding 0.56% and 0.68% Met (*P* < 0.05). In vaccinated chickens, CD8^+^ cell percentages were reduced after feeding 0.68% Met (*P* < 0.05). The CD4^+^/CD8^+^ was not influenced by Met concentrations in the diet in medicated chickens (*P* > 0.05), but decreased by high diet Met concentrations in vaccinated chickens (*P* < 0.05).

**Table 6:** Blood immune cell percentages, cecal luminal sIgA contents and the mRNA expression of cytokines in cecal tonsils after indicated treatments^1^

	Medicated groups			Vaccinated groups			*P* value		
Met^2^ concentrations:	0.45%	0.56%	0.68%	0.45%	0.56%	0.68%	Anticoccidial program	Met levels	Anticoccidial program × Met levels
CD4 (%)	9.72 ± 1.05^c^	12.27 ± 1.36^a^	12.12 ± 1.33^a^	12.95 ± 1.42^a^	10.85 ± 1.27^b^	10.61 ± 1.17b^c^	0.092	0.028	0.002
CD8 (%)	8.51 ± 0.92^d^	11.16 ± 1.23^a^	10.96 ± 1.08^ab^	10.24 ± 1.14^b^	9.51 ± 1.03^bc^	9.21 ± 0.99^c^	0.045	0.015	0.005
CD4/CD8	1.15 ± 0.11^b^	1.10 ± 0.11^b^	1.11 ± 0.13^b^	1.27 ± 0.12^a^	1.14 ± 0.12^b^	1.16 ± 0.13^b^	0.023	0.041	0.003
sIgA (mg/mL)	1.30 ± 0.19^b^	1.47 ± 0.22^a^	1.52 ± 0.27^a^	1.50 ± 0.24^a^	1.49 ± 0.21^a^	1.50 ± 0.28^a^	0.028	0.037	0.008
*TNF-α*	1.09 ± 0.11^b^	1.39 ± 0.20^a^	1.47 ± 0.26^a^	0.83 ± 0.09^c^	0.87 ± 0.10^c^	0.81 ± 0.07^c^	0.008	0.035	0.025
*IL-2*	1.04 ± 0.09^b^	1.27 ± 0.14^a^	1.23 ± 0.18^a^	0.97 ± 0.11^b^	0.95 ± 0.09^b^	0.98 ± 0.07^b^	0.013	0.039	0.031
*IFN-γ*	1.10 ± 0.14^e^	1.54 ± 0.25^c^	1.62 ± 0.29^b^	1.76 ± 0.33^a^	1.33 ± 0.22^d^	1.14 ± 0.13^e^	0.074	0.005	0.001

^1^An anticoccidial drug (narasin, 60 mg/kg diet) was added to diets in medicated groups, and an anticoccidial 7-valent live vaccine was inoculated in vaccinated groups

^2^*Met* methionine, *CD4* CD4+ cells, *CD8* CD8+ cells, *sIgA* secreted immunoglobulin A, *TNF-α* tumor necrosis factor-α, *IL-2* interleukin-2, *IFN-γ* interferon-γ

^a,b,c,d,e^The data of the same row not sharing a lowercase letter differ (*P* < 0.05, n = 30)

Feeding 0.56% and 0.68% Met was associated with elevated sIgA level in medicated chickens (*P* < 0.05), but had no effect on sIgA level in vaccinated chickens (*P* > 0.05). When Met concentrations in the diet for medicated chickens were increased to 0.56 and 0.68%, *TNF-α* and *IL-2* mRNA expression levels were dramatically increased (*P* < 0.05). mRNA expression of *TNF-α* and *IL-2* in vaccinated chickens was not affected by dietary Met concentrations. *IFN-γ* mRNA expression was increased in medicated chickens, but decreased in vaccinated chickens, with Met level increased to 0.56% and 0.68% (*P* < 0.05). Feeding 0.45% Met to vaccinated chickens had the highest *IFN-γ* mRNA expression among all treatments.

### The serum anti-oxidative parameters of chickens

As indicated in Table 7, anticoccidial programs had no effect on serum MDA concentration (*P* > 0.05), but impacted on GPx activity and T-AOC (*P* < 0.05). Dietary Met levels affected all these indexes (*P* < 0.05). There were interactions between anticoccidial programs and dietary Met levels for GPx activity and T-AOC (*P* < 0.05). Serum GPx activity in medicated chickens was increased by feeding 0.56% Met (*P* < 0.05), compared to 0.45% Met. But the beneficial effect was not evident by feeding 0.68% Met (*P* > 0.05). High Met levels in diets did not affect serum GPx activity in vaccinated chickens (*P* > 0.05).

**Table 7:** The serum anti-oxidative parameters after indicated treatments^1^

	Medicated groups			Vaccinated groups			*P* value		
Met^2^ concentrations:	0.45%	0.56%	0.68%	0.45%	0.56%	0.68%	Anticoccidial program	Met levels	Anticoccidial program × Met levels
GPx (U/mL)	1285.26 ± 172.80^b^	1392.71 ± 194.74^a^	1321.81 ± 186.24^ab^	1240.43 ± 157.82^b^	1266.49 ± 160.72^b^	1265.93 ± 161.22^b^	0.042	0.038	0.021
MDA (nmol/mL)	6.93 ± 0.72^e^	8.62 ± 0.89^c^	10.64 ± 1.11^a^	7.74 ± 0.99^d^	8.66 ± 1.19^c^	9.40 ± 1.34^b^	0.102	0.015	0.207
T-AOC (mmol Trolox)	9.55 ± 1.07^b^	10.36 ± 1.96^a^	9.85 ± 1.53^ab^	8.46 ± 0.93^c^	8.77 ± 1.01^bc^	8.79 ± 1.17^bc^	0.023	0.032	0.035

^1^An anticoccidial drug (narasin, 60 mg/kg diet) was added to diets in medicated groups, and an anticoccidial 7-valent live vaccine was inoculated in vaccinated groups

^2^*Met* methionine, *GPx* glutathione peroxidase, *MDA* malondialdehyde, *T-AOC* total antioxidant capacity

^a,b,c,d,e^The data of the same row not sharing a lowercase letter differ (*P* < 0.05, *n* = 30)

Compared to 0.45% Met, feeding 0.56 and 0.68% Met increased MDA in serum to 8.62 (*P* < 0.05) and 10.84 nmol/mL (*P* < 0.01) from 6.93 nmol/mL in medicated chickens, and to 8.66 (*P* < 0.05) and 9.40 nmol/mL (*P* < 0.05) from 7.74 nmol/mL in vaccinated chickens. Serum T-AOC in medicated chickens was enhanced by feeding 0.56% Met (*P* < 0.05), but not by feeding 0.68% Met (*P* > 0.05). High Met levels in diets did not affect T-AOC in serum in vaccinated chickens (*P* > 0.05).

## Discussion

Coccidiosis is a major parasitic disease of poultry, associated with malabsorption, inefficient feed utilization, impaired growth rate and increased mortality in broilers. *E. tenella* is one of seven species of coccidia that infect chickens. After the oocysts are ingested by chickens, they experience the excystation to generate the invasive sporozoites, which penetrate into epithelial cells of the intestinal mucosa, often causing serious damage to the physical integrity and normal functions of the gut particularly in domestic chickens [16]. Previous study demonstrated that coccidial challenge reduced the amounts of L-Met absorption of broilers by 52% compared to unchallenged controls [17]. Anticoccidial drugs are extensively adopted to combat coccidial infection in the poultry industry, but their influences on Met absorption and requirement of growing broilers challenged by *E. tenella* are seldom investigated. In the present study, chicks were challenged with *E. tenella* by oral gavage of the sporulated oocysts, and were also provided diets containing narasin to control coccidiosis. In this case, feeding increased Met (0.56% and 0.68%) to medicated chicks improved the growth performance compared to 0.45% Met. Previous reports showed that feeding increased Met either had no effects or decreased the growth performance of chicks that were not challenged by coccidia [18, 19]. In general, 1.2% Met is a tolerable excess level that would not depress voluntary food intake or growth rate of broilers [20]. Increasing dietary Met level beyond this value linearly decreased weight gain, feed intake, and feed efficiency of chickens, which is probably due to the toxicity of the sulfur amino acids [21, 22]. Narasin itself has no effect on production performance and nutrient utilization in broiler chickens [23]. Our data, thus, suggest an increased Met requirement of broiler chickens given narasin under *E. tenella* -challenged conditions.

There is increasing concern about rising levels of drug resistance of *Eimeria* species. Narasin is an ionophore coccidiostat, processing a potent, broad-spectrum anticoccidial activity against *Eimeria* species. The control mechanism of ionophore coccidiostats is altering the ion transport and disrupting osmotic balance, which can damage the coccidial sporozoites during their release into the lumen [24]. However, the extensive and long-term use in chemotherapy to control coccidiosis leads to the generation of drug resistance. Peek and Landman [25] found that one isolated *E. tenella* from Dutch broilers in 1996 was sensitive to narasin, however, two isolated *E. tenella* in 1999 showed resistance to narasin. Another study found that replacing a proportion of nicarbazin with narasin results in reduced sensitivity to most coccidial species [26]. Due to drug resistance, some *E. tenella* probably still survive after the exposure to narasin and cause malabsorption and inefficient feed utilization of chickens. This may partly explain the increased Met requirement of the medicated broiler chickens under *E. tenella*-challenged conditions. In addition, we found that feeding increased Met to medicated chickens improved small intestinal morphology, as indicated by the improved VH, CD and V/C as well as reduced cecal lesion and fecal oocyst output. Immunological assays further revealed that immune functions of chickens fed anticoccidial drug were enhanced with the increase of Met levels in diet. These data suggested that increasing Met levels in diet helped medicated chickens antagonize the challenge of *E. tenella* through the stimulation of immune functions. Met is known to play an important role in the immune functions by regulating antibody production and cell-mediated immune responses. We hypothesized that anticoccidial drug narasin may be incapable of most effectively controlling the number of *E. tenella* probably due to the drug resistance under a heavy load of *E. tenella* infection. Many survived *E. tenella* induced the host immune response that is conducive to eliminating these pathogenic micro-organisms. Increased Met in diet just meets the increased requirement for the stimulated immune functions. Following exposure to *Eimeria* species either naturally or via vaccination, chicks gradually develop acquired immunity and become resistant to reinfection of coccidia [9, 16]. *Eimeria* infection can induce a significant increase of CD8^+^ cells in the intraepithelial leucocytes, tonsil lymphocytes and peripheral blood [27–30]. CD4^+^ cells were also increased in the intraepithelial leucocytes, but decreased in the peripheral blood and spleen [29, 31]. The decrease in CD4+ cells was observed after primary infection of *Eimeria*, but not the secondary infection [29, 31]. To understand the role of these T cells in the protection against coccidiosis, previous experiments studied the response of immunosuppressed chickens by elimination of specific T cells to coccidial challenge. The selective depletion of CD4^+^ cells reduced *IFN-γ* mRNA expression in the cecal tonsil and intraepithelial lymphocytes and increased production of oocysts of *E. tenella* in chicks [28, 32], which suggests the important role of CD4^+^ lymphocytes in controlling primary infection with *E. tenella.* The selective elimination of CD8^+^ cells resulted in the exacerbation of disease as evidenced by increased oocyst shedding after secondary infection with *E. tenella* or *E. acervulina* [32]*.* It is known that CD4^+^ cells play an important helper function in protective immune responses, and CD8^+^ cells act as effector cells in these processes [33]. Both CD4+ and CD8+ cells are responsible for the IFN-γ production [33]. IFN-γ production is higher in chicks infected with *Eimeria* species compared with non-infected chicks, and higher levels of IFN-γ coincide with a protective immune response to the infection [27]. Previous study found that pretreatment with cultural supernatants containing IFN-γ inhibited the intracellular development of *E. tenella* [34, 35]. Apart from IFN-γ, factors like IL-2 and IgA are induced for the resistance to *E. tenella,* as reviewed by Lillehoj et al. [35]. Although coccidial vaccination confers the protection of the host from coccidial infection, the enhanced immune functions likely increase Met consumption for the synthesis of more immunity-related proteins. Thus, it is necessary theoretically to increase Met supply to meet this specific requirement. Unexpectedly, in the present study, feeding a diet containing 0.56% Met had no growth stimulating effect on vaccinated chickens, compared to 0.45% Met. The increment of Met to 0.68% conversely decreased the feed intake and weight gains. In addition, we found that supplementation with high Met levels decreased CD4^+^ and CD8^+^ cells in the blood and suppressed *IFN-γ* mRNA expression in cecal tonsils, suggesting an inhibited immune function against *E. tenella*. Maybe the reason is that coccidial vaccination confers the protection of the host from coccidial infection, so 0.45% Met is sufficient for chickens vaccinated. Excess Met could be detrimental to the anti-oxidative system and immune function. It was reported that feeding Met-sufficient diets (containing 0.50 and 0.70% Met) decreased the growth, feed intake and efficiency of feed utilization of chickens that were injected with some components of microbial pathogens, like *Escherichia coli* lipopolysaccharide or heat-killed *Staphylococcus aureus* [36]. This study suggests that Met-sufficient diet contributes to the generation of immunologic stress upon the stimulation by the immunogens. In the status of immunologic stress, increased releases of cytokines, including IL-1 and TNF-α, are associated with increased production of adrenocorticotropin and thyroxin-stimulating hormones. These metabolism-related hormones may be implicated in the poor growth performance. There is evidence indicating that TNF-α plays biphasic roles following *E. tenella* infection. TNF-α leads to the development of a protective immunity on the one hand, but, on the other hand, it participates in the pathogenesis of coccidiosis, because treatment of chickens with antibodies against TNF-α results in a partial abrogation of *E. tenella*-induced body weight loss in chickens [35]. Although *TNF-α* mRNA expression in cecal tonsils of the vaccinated chickens was not affected by increased diet Met levels, it was unclear whether the influence on TNF-α occurs in other immune organs, such as the spleen and intraepithelial lymphocytes. Further studies are warranted to investigate TNF-α alterations in these locations.

*Eimeria* infection increases the activity of NADPH oxidase, an important enzyme for production of oxidative species in infected mucosa [37]. It is known that Met serves as a precursor for the synthesis of GSH and taurine and hence plays a role in the defense against oxidative stress. It has reported that feeding 0.5% Met promotes the synthesis of the GSH and GPx activity in the liver of growing chicks co-fed increased unsaturated fats [38]. Broilers given a diet containing 0.59% Met have a higher hepatic GSH: GSH disulphide ratio than those given a diet containing 0.49% Met at day 21. In addition, broilers given high dietary Met have enhanced serum superoxide dismutase activity and decreased MDA content at day 7 [39]. Wen et al. [40] found that feeding 0.57% Met increases T-AOC in breast muscle of fast-growing broilers compared to 0.44% Met. These studies suggest that a higher dietary Met concentration improves antioxidant status in broilers. Under heat stress conditions, feeding high levels of Met to broilers contributes to increased expression levels of genes related to antioxidant activity, including cystathionine β-synthase, GSH synthetase and GPx [41]. In the present study, increasing dietary Met concentrations from 0.45% to 0.56% was associated with increased GPx activity and T-AOC in serum in medicated chickens. But, the increase in dietary Met concentrations had no such effect in vaccinated chickens. MDA is one of the final products of polyunsaturated fatty acids peroxidation. As polyunsaturated fatty acids are highly susceptible to oxidative species compared with other macromolecular substances, such as protein and DNA, MDA is considered as a sensitive biomarker of oxidative stress. We found that dietary Met increased serum MDA levels in both medicated and vaccinated chickens in a dose-dependent manner. It is likely that feeding high levels of Met increases production of oxidative species in chickens under specific conditions, although Met can contribute to the synthesis of antioxidant products and enzymes.

## Conclusions

In the present study, vaccinated chickens that were given 0.45% Met showed greater growth performance than medicated chickens that were provided basal (0.45%) or even increased levels of Met. Therefore, inoculation with anticoccidial vaccine is a better strategy for controlling coccidiosis than feeding narasin, not only due to greater growth performance obtained by the vaccination, but also due to the lower Met supplementation to achieve optimized performance. Furthermore, the present study for the first time revealed that high dietary Met levels benefit the growth performance of mediated chickens under *E. tenella*-challenged condition, but has no beneficial effects on that of vaccinated chickens.

## Abbreviations

ADFI: Average daily feed intake
ADG: Average daily gain
CD: Crypt depth
*E. acervulina*: *Emeria acervulina*
*E. tenella*: *Emeria tenella*
F/G: Feed to gain ratio
GPx: Glutathione peroxidase
GSH: Glutathione
IFN-γ: Interferon-γ
Ig: Immunoglobulin
IL-2: Interleukin-2
MDA: Malondialdehyde
Met: Methionine
ON: Oocyst output
PBS: Phosphate-buffered saline
T-AOC: Total antioxidant capacity
TNF-α: Tumor necrosis factor-α
V/C: VH to CD ratio
VH: Villus height
